# Wasabi Gone Wild? Origin and Characterization of the Complete Plastomes of Ulleung Island Wasabi (*Eutrema japonicum*; Brassicaceae) and Other Cultivars in Korea

**DOI:** 10.3390/genes15040457

**Published:** 2024-04-05

**Authors:** JiYoung Yang, Cheon Gyoo Park, Myong-Suk Cho, Seung-Chul Kim

**Affiliations:** 1 Research Institute for Dok-do and Ulleung-do Island, Kyungpook National University, 80 Daehak-ro, Buk-gu, Daegu 41566, Republic of Korea; jyyangson@knu.ac.kr; 2 Gangwondo Agricultural Research and Extension Services, Wild Vegetable Reseaerch Institute, Alpine Agricultural Experiment Station, Taebaek-si 26046, Republic of Korea; politefrog@korea.kr; 3 Department of Biological Sciences, Sungkyunkwan University, 2066 Seobu-ro, Suwon 16419, Republic of Korea; marina0426@gmail.com

**Keywords:** *Eutrema*, Brassicaceae, chloroplast genome, chlorotype, Ulleung Island, wasabi cultivars

## Abstract

Korean wasabi occurs naturally on the young oceanic, volcanic Ulleung Island off the east coast of the Korean Peninsula. Although the Ulleung Island wasabi is reported as *Eutrema japonicum* and has been suggested to be morphologically identical to cultivars in Korea, very little is known about its taxonomic identity and relationship with other cultivars. In this study, we sequenced the complete chloroplast DNA sequences of three naturally occurring Ulleung Island wasabi plants and six cultivars (‘Daewang’, ‘Daruma’, ‘Micado’, ‘Orochi’, ‘Green Thumb’, and ‘Shogun’) from continental Korea and determined the taxonomic identity of Korean wasabi on Ulleung Island. The size and organization of the complete chloroplast genomes of the nine accessions were nearly identical to those of previously reported wasabi cultivars. In addition, phylogenetic analysis based on the complete plastomes suggested that Ulleung Island wasabi most likely comprises various wasabi cultivars with three chlorotypes (‘Shogun’, ‘Green Thumb’, and a unique Chusan type). Based on the complete plastomes, we identified eight chlorotypes for the major wasabi cultivars and the Ulleung Island wasabi. Two major groups (1—‘Mazuma’ and ‘Daruma’, and 2—‘Fujidaruma’/‘Shimane No. 3’/Ulleung Island wasabi/five cultivars in Korea) were also identified based on mother line genealogical history. Furthermore, different types of variations (mutations, insertions/deletions (indels), mononucleotide repeats, and inversions) in plastomes were identified to distinguish different cultivar lines and five highly divergent hotspots. The nine newly obtained complete plastomes are valuable organelle genomic resources for species identification and infraspecific phylogeographic studies on wild and cultivated wasabi.

## 1. Introduction

Of the three valuable Cruciferae condiments in the Japanese diet (i.e., wasabi, grated radish, and mustard), wasabi (*E. japonicum* = *Wasabia japonica*) plays a vital role in Japanese cuisine and culture [1,2,3,4]. Wasabi, a perennial herb with an enlarged and pungent stem, has been widely used in Japanese foods, such as raw sliced fish (sushi or sashimi) and noodle dishes (e.g., soba) [2]. The earliest record of wasabi as an herb can be traced back to the sixth or early seventh century in central east Japan [5]. Wasabi belongs to the genus *Eutrema* of the mustard family Brassicaceae, and comprises approximately 26 species, most of which are distributed in East Asia, including *E. japonicum* (wasabi) and *E. tenue* (yuriwasabi) [6]. While *E. japonicum* occurs naturally from Russia’s Sakhalin Island (north of Hokkaido, the northernmost island) to Kyushu (the southernmost island in Japan) and includes several cultivars and escaped cultivars, *E. tenue* is found only in Japan [2]. These two wild wasabi species can be distinguished based on cytology and vegetative morphology (i.e., stem size and color, and leaf size and shape) [7,8,9]. A previous study suggested that *E. yunnanense* from China is closely related to two Japanese *Eutrema* species, especially *E. japonicum,* based on morphological similarity [2,10]. Similar to *E. tenue* in Japan, which is known only in the wild, an ethnobotanical survey suggested no evidence of domestication of *E. yunnanense* in China [2]. Furthermore, a molecular phylogenetic study demonstrated that the three species (*E. japonicum*, *E. tenue*, and *E. yunnanense*) formed a clade and were clearly separated from other Brassicaceae species [2]. However, the two Japanese species are highly differentiated from the Chinese *E. yunnanense*, with an estimated divergence time of five million years ago (Mya) [2].

Although the full domestication history of *E. japonicum* remains elusive, numerous wasabi cultivars have been developed and described [11]. Wasabi cultivars are considered regionally specific in Japan and often named either for their region of cultivation (e.g., ‘Shimane 3’ for Shimane Prefecture) or for the person who developed it (e.g., ‘Izawa Daruma’ for K. Izawa), and the Shimane region has the largest collective area of wasabi production in Japan [9]. Japanese farmers commonly practice vegetative propagations (usually for up to three generations) and cross-pollination mediated seedling selection to overcome diseases and production problems [12]. The cross-pollination of local cultivars—also known as “cultivated mutant” [13]—has raised concerns about their purity [14]. In addition, lineage archiving over the long history of wasabi cultivation by farmers has often been incomplete, so tracing the origins and relationships among modern cultivars is sometimes difficult. Nevertheless, approximately twenty modern wasabi cultivars are commonly used, and it has been suggested that three major cultivars, ‘Fujidaruma’, ‘Shimane No. 3’, and ‘Mazuma’, played an important role in modern cultivar developments [3,15]. The complete chloroplast genome sequences were highly effective in determining the evolutionary history of *Eutrema*: (1) non-monophyly of wasabi and yuriwasabi, (2) the estimated divergence time of 1.3 Mya for the Japanese *Eutrema* (two wild species, *E. japonica* and *E. tenue* from Fukuoka, and *E. japonica* native variety and cultivars) from the Chinese *E. yunnanense* and *E. tenue* (yuriwasabi; Gifu) clade, and (3) the development of DNA markers to discriminate among cultivars [3].

**Figure 1:**
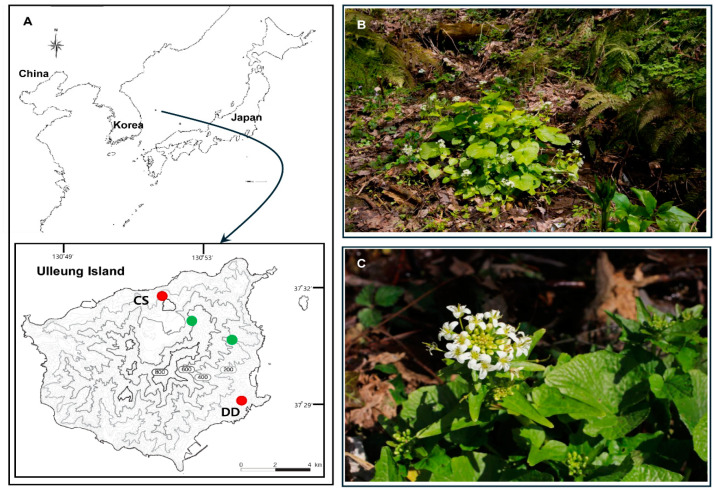
(**A**) A map showing the location of Ulleung Island, Korea, with four commonly known locations of the wasabi population shown in green and red dots. Two red dot populations (Chusan, CS and Dodong, DD) were sampled in this study. Photographs of the Ulleung Island wasabi, showing a typical wet habitat (**B**), with flowers (**C**). Photo credit: Young-Bong Park.

Ulleung Island, an oceanic volcanic island between the Korean Peninsula and the Japanese Archipelago, is approximately 1.8 million years old [16,17] (Figure 1A). The flora is rich with approximately 500 native vascular plant species, approximately 40 (ca. 8%) of which are endemic to Ulleung Island [18]. The island is known for its exceptionally high level of anagenetic speciation, and nearly 90% of all endemic plants are anagenetically derived [19,20]. Several recent studies have taken advantage of the unique settings on Ulleung Island and investigated the anagenetic speciation mechanism of insular plant endemics (e.g., *Rubus takesimensis* [21]; *Campanula takesimana* [22]; *Phedimus takesimensis* [23]; *Prunus takesimensis* [24]). Several floristic studies have reported the occurrence of wasabi plants on Ulleung Island, Korea [25,26,27] (Figure 1B,C). Although Ulleung Island wasabi plants have been reported as *E. japonicum* and are suggested to be morphologically identical to cultivars in Korea [18,28,29,30], molecular insights into their taxonomic identity are very limited. Even if the Ulleung Island wasabi was originally introduced into cultivation and has now escaped and naturalized around the island, we still know very little about its cultivar identity and number of existing cultivars on the island. Alternatively, wasabi plants could be native to Ulleung Island. The only available molecular marker study based on random amplified polymorphic DNA (RAPD), which included one individual from Ulleung Island wasabi, was inconclusive in determining its cultivar type and relationships with other modern cultivars [31].

Therefore, we assembled and characterized the complete chloroplast genome sequences of naturally occurring Ulleung Island wasabi plants and compared them to several other major cultivars currently available in Korea. In addition, by conducting phylogenetic analysis based on complete plastome sequences, including previously sequenced wild wasabi (*E. japonicum*) and yuriwasabi (*E. tenue*) in Japan, we hoped to determine whether the naturally occurring Ulleung Island wasabi plants are the wild type, and, if not, which cultivars are represented on Ulleung Island. Newly assembled plastomes of wasabi cultivars could be used to build a chloroplast genomic reference for wasabi cultivars and identity DNA markers to discriminate among them.

## 2. Results

### 2.1. Genome Size and Features

**Figure 2:**
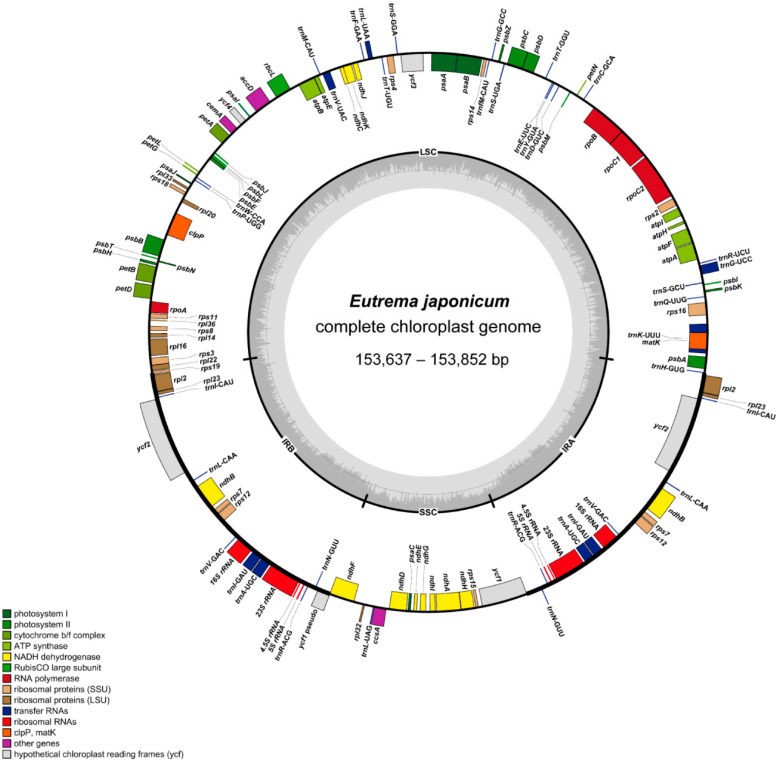
Complete plastome map of three putative wild Ulleung Island wasabi and six cultivar accessions in Korea. The genes located outside of the circle are transcribed counterclockwise, while those located inside are transcribed clockwise.

We characterized three putative wild Ulleung Island wasabi accessions (Kicheongsan, Dodong, and Chusan) and six cultivars (‘Daruma’, ‘Shogun’, ‘Green Thumb’, ‘Daewang’, ‘Micado’, and ‘Orochi’) at Gangwondo Agricultural Research and Extension Services (GARES), Korea. The length of the complete plastome sequences ranged from 153,794 bp (‘Daruma’) to 153,852 bp (Dodong and ‘Shogun’), and the plastomes were highly conserved with no structural variation or content rearrangements (Figure 2 and Table 1). The large single-copy (LSC) region, small single-copy (SSC) region, and two inverted repeat (IR) regions ranged from 83,889 bp (Kicheongsan) to 84,006 bp (seven accessions excluding ‘Daruma’), from 17,790 bp (‘Daruma’) to 17,812 bp (‘Shogun’), and 26,007 bp (‘Daruma’) to 26,017 bp (seven accessions excluding ‘Micado’), respectively (Table 1). The plastomes of three Ulleung Island wasabi samples and six cultivars from GARES contained 131 genes, including 84 protein-coding genes, 8 rRNA, 37 tRNA genes, and their overall guanine–cytosine (GC) content was identical (36.4%). All plastomes contained 17 duplicated genes in the IR regions, including seven tRNA, four rRNA, and six protein-coding genes. Fifteen genes (*ndhA*, *ndhB*, *petB*, *petD*, *rpl2*, *rpl16*, *rpoC1*, *rps12*, *rps16*, *trnA*-UGC, *trnG*-UCC, *trnI*-GAU, *trnK*-UUU, *trnL*-UAA, and *trnV*-UAC) contained one intron, whereas *clpP* and *ycf3* contained two introns. The complete *ycf1* in all nine plastomes was positioned in the SSC/IRa junction with a length of 5379 bp, whereas the pseudogenized *ycf1* was positioned in the IRb/SSC junction region with a length of 1128 bp.

**Figure 3:**
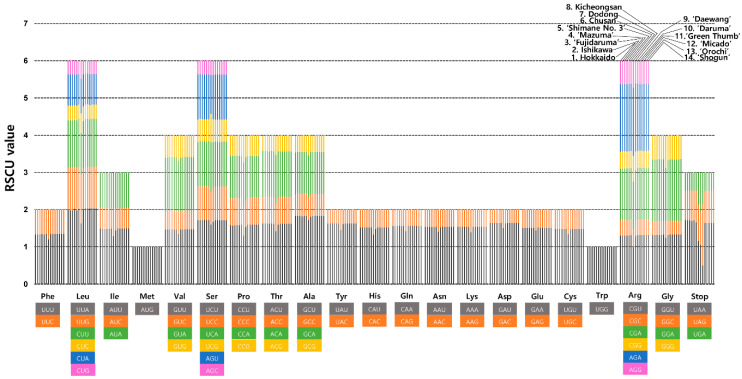
Relative Synonymous Codon Usage (RSCU) in Ulleung Island wasabi accessions, three major Japanese cultivars, and six cultivars at GARES. The list of accessions from left to right columns represent Japanese wild wasabi (1. Hokkaido, 2. Ishikawa), three major Japanese cultivars (3. ‘Fujidaruma’, 4. ‘Mazuma’, and 5. ‘Shimane No. 3’), Ulleung Island wasabi (6. Chusan, 7. Dodong, and 8. Kicheongsan), and major cultivars at GARES (9. ‘Daewang’, 10. ‘Daruma’, 11. ‘Green Thumb’, 12. ‘Micado’, 13. ‘Orochi’, and 14. ‘Shogun’).

The frequency of codon usage of the six wasabi plastomes from GARES and the three from Ulleung Island, along with the three major Japanese cultivars (‘Fujidaruma’, ‘Mazuma’, and ‘Shimane No. 3’), and the two wild Japanese wasabi plastomes (Hokkaido and Ishikawa) of *E. japonicum* was calculated based on the protein-coding sequences (Figure 3). The results showed that the average codon usage among the 14 accessions ranged from 26,184 (Ulleung Island wasabi Kicheongsan) to 26,637 (‘Mazuma’) (Supplementary Table S1). The average codon usage for the remaining accessions was 26,134 for Hokkaido wild wasabi, 26,136 for ‘Fujidaruma’, Ishikawa, ‘Shimane No. 3’, 26,200 for ‘Daewang’, ‘Daruma’, ‘Green Thumb’, ‘Micado’, ‘Orochi’, and ‘Shogun’, and 26,227 for two Ulleung Island wasabi (Chusan and Dodong). The highest relative synonymous codon usage (RSCU) value was indicated in the usage of UUA codon for leucine (1.63–2.03) followed by GCU for alanine (1.72–1.83) and UCU for Serine (1.6–1.72). The lowest RSCU value was indicated in the usage of CUG for leucine (0.35–0.49), AGC for serine (0.36–0.39), and GAC for aspartic acid (0.36–0.49) (Supplementary Table S1). All relative synonymous codon usage (RSCU) values of Ulleung Island Dodong accession showed slightly different values when compared to the others. Leucine usage in particular was confirmed to be 1.63, 1.31, 1.12, 0.53, 0.92, and 0.49 for the codons UUA, UUG, CUU, CUC, CUA, and CUG, respectively; the other accessions showed 1.9–2.03, 1.1–1.15, 1.27–1.29, 0.38–0.4, 0.83–0.85, and 0.35–0.38, respectively. The usage of UCU, UCC, UCA, UCG, AGU, AGC for serine was shown to be 1.6, 0.88, 1.34, 0.75, 1.05, and 0.39, while the other accessions were 1.68–1.72, 0.9–0.91, 1.2–1.24, 0.59–0.61, 1.1–1.21, and 0.36–0.38, respectively. The usage of the CGU, CGC, CGA, CGG, AGA, AGG codons for arginine was shown to be 1, 0.47, 1.29, 0.68, 1.84, and 0.71, while the other accessions were 1.26–1.32, 0.41–0.42, 1.38–1.39, 0.46–0.51, 1.79–1.81, and 0.61–0.63, respectively. Furthermore, it was confirmed that UAA, UAG, and UGA were used for stop codons in 1.15, 0.98, and 0.86, whereas the other accessions were 1.64–1.71, 0.8–1.64, and 0.48–0.5, respectively. Additionally, UAA, UAG, and UGA were used as stop codons, with Kicheongsan using 1.06, 0.98, and 0.86 and ‘Daewang’ accession using 0.5, 1.64, and 0.86, respectively.

### 2.2. Comparative Analysis of Genome Structure

**Figure 4:**
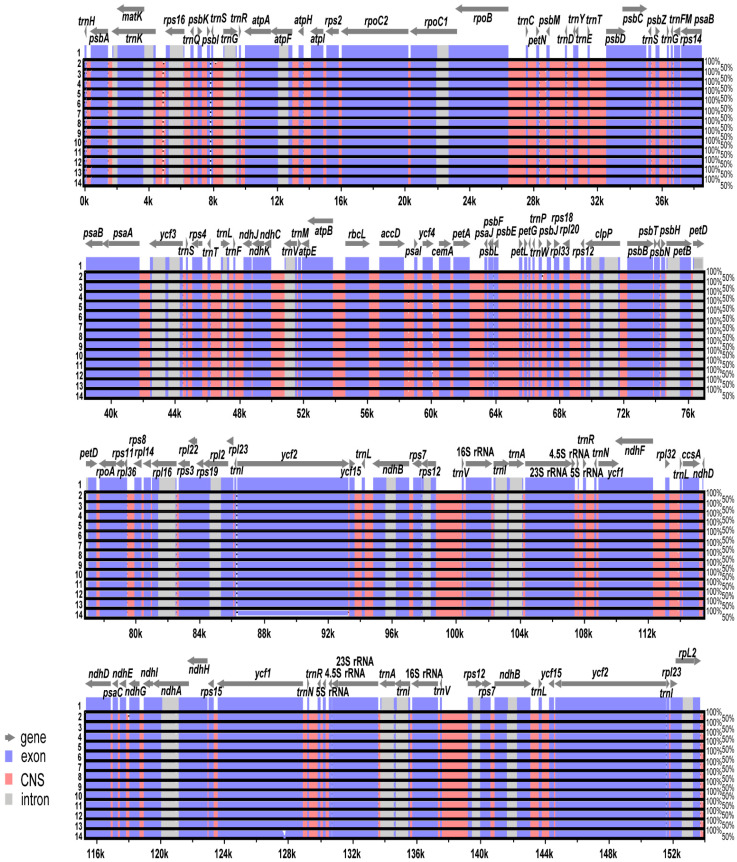
Visualization of alignment of the 14 plastome sequences of *E. japonicum* accessions. Accessions: Japanese wild (1. Hokkaido; 2. Ishikawa); Japanese cultivar (3. ‘Fujidaruma’; 4. ‘Mazuma’; 5. ‘Shimane No. 3’); Ulleung Island wasabi (6. Chusan; 7. Dodong; 8. Kicheongsan); GARES cultivars (9. ‘Daewang’; 10. ‘Daruma’; 11. ‘Green Thumb’; 12. ‘Micado’; 13. ‘Orochi’; 14. ‘Shogun’).

**Figure 5:**
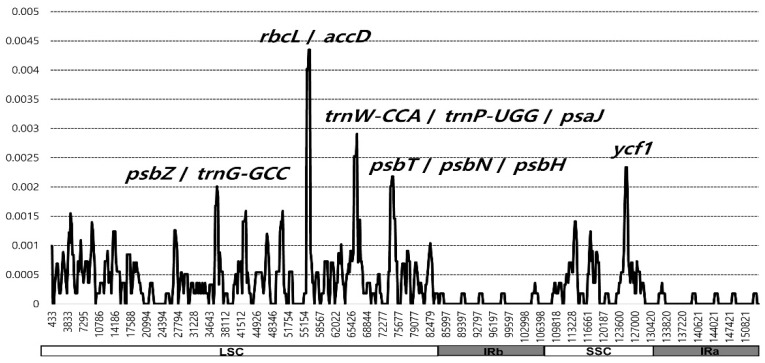
Sliding window analysis of the 14 complete chloroplast genomes of *E. japonicum* accessions. The vertical and horizontal axes are nucleotide diversity (Pi) and position of the window midpoint, respectively.

The 14 complete plastome sequences of *E. japonicum* accessions were plotted using mVISTA analysis [32], with the annotated wild Hokkaido plastome as a reference (Figure 4). The results suggest that most regions were highly conserved, and very little sequence variations existed within *E. japonicum* plastomes. Sliding windows analysis using the DnaSP program [33] revealed highly variable regions in 14 accessions of *E. japonicum* plastomes (Figure 5). The average value of nucleotide diversity (Pi) over the entire complete chloroplast genome was 0.000342. The most variable region was the *rbcL*/*accD* intergenic region with a Pi value of 0.00454. In addition, the highly variable regions included four intergenic regions and one genic region: *psbZ*/*trnG*-GCC (Pi = 0.00201), *psbT*/*psbN*/*psbH* (Pi = 0.00218), *trnW*-CCA/*trnP*-UGG/*psaJ* (Pi = 0.00218), and *ycf1* (Pi = 0.00234). Therefore, five highly variable region with Pi value greater than 0.00201, which can be used for population genetics and phylogeographic studies within *E. japonicum*, were identified in the 14 *E. japonicum* plastomes.

We identified two major groups among the three major cultivars from Japan, three Ulleung Island wasabi, and six cultivars from GARES based on the patterns of mutations, insertions/deletions (indels), and inversions in the chloroplast genomes (Supplementary Table S2). The first group included the two major cultivars of ‘Mazuma’ and ‘Daruma’ and the second one included the remaining major cultivars and the three samples of wasabi from Ulleung Island, i.e., two Japanese cultivars (‘Fujidaruma’ and ‘Shimane No. 3’), three Ulleung Island wasabi (Chusan, Dodong, and Kicheongsan), and five other cultivars (‘Daewang’, ‘Green Thumb’, ‘Micado’, ‘Orochi’, and ‘Shogun’). These two major groups of cultivars were distinguished by 54 point mutations, 18 indels, 11 polyT/C’s, and 2 inversions (11 and 4 bp). The following plastomes were identical: (1) Dodong and ‘Shogun’, (2) ‘Micado’ and ‘Orochi’, (3) Kicheongsan, ‘Daewang’, and ‘Green Thumb’. Based on the variable sites of the major wasabi cultivars from Japan and GARES, and the three samples of wasabi from Ulleung Island, eight complete plastome genotypes (i.e., chlorotypes) were found: (1) Dodong-‘Shogun’, (2) ‘Micado’-‘Orochi’, (3) Kicheongsan-‘Daewang’-‘Green Thumb’, (4) Chusan, (5) ‘Shimane No. 3’, (6) ‘Fujidaruma’, (7) ‘Mazuma’, and (8) ‘Daruma’. We confirmed two variable sites between ‘Shimane No. 3’ and ‘Fujidaruma’ identified by Haga et al. [3], i.e., one point mutation (C/T in *rpoC2*) and one poly T’s (10T’s/9T’s in *rpl32*/*trnL*-UAG intergenic region).

### 2.3. Phylogenetic Analysis

**Figure 6:**
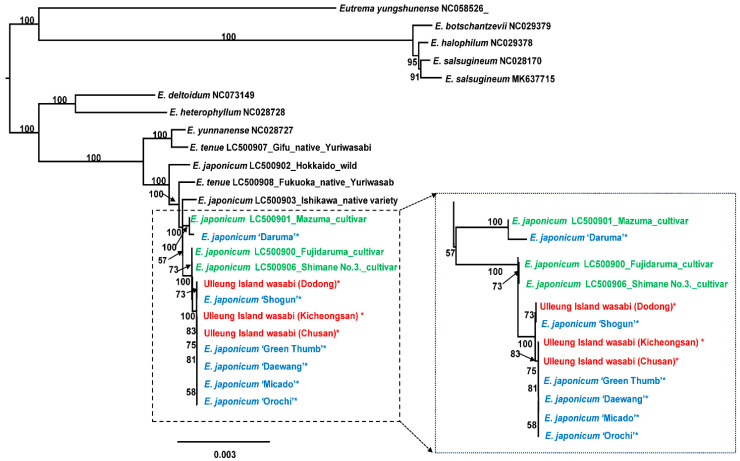
The maximum-likelihood (ML) tree inferred from fourteen accessions of *E. japonicum* and ten congeneric *Eutrema* species plastomes. The bootstrap value based on 1000 replicates is shown on each node. Nine newly sequenced accessions in the current study are indicated using asterisks. The accessions in green, blue, and red are major cultivars from Japan, cultivars from GARES, and three samples of wasabi from Ulleung Island, respectively.

Maximum likelihood (ML) analysis was conducted using the best-fit model of TVM+F+R3. A total of 130,594 aligned nucleotide bases contained 2607 parsimony-informative sites. The ML tree confirmed previously reported phylogenetic relationships among wild wasabi species [3]. Within the ingroup *Eutrema*, two major lineages were recovered: the one including *E. deltoidum* and *E. heterophyllum* and the other including *E. yunnanense*, *E. tenue*, and *E. japonicum* (wild, native variety, and major cultivars) from Japan and GARES, along with three putative wild samples from Ulleung Island (Figure 6). As shown previously [3], yuriwasabi *E. tenue* is not monophyletic; one accession (LC500907; Gifu native yuriwasabi) is sister to Chinese *E. yunnanense*, while the other is sister to the clade containing wild and cultivated wasabi *E. japonicum* sampled from Japan and Ulleung Island, Korea. However, the status of this species, based on plastome relationship, is uncertain and requires more detailed research.

In terms of relationships between wild and cultivated *E*. *japonicum*, the Ishikawa native variety (LC500903) was sister to the clade containing all the cultivars sampled in this study. This study determined the identity of the putative wild accessions of wasabi from Ulleung Island, Korea, for the first time. Of the three accessions we sampled in this study, the Kicheongsan accession is sister to the clade containing Chusan and four cultivars (‘Daewang’, ‘Green Thumb’, ‘Micado’, and ‘Orochi’). In addition, the plastomes of Dodong and ‘Shogun’ are identical, while the Chusan accession is sister to the clade containing four cultivars, i.e., ‘Daewang’, ‘Green Thumb’, ‘Micado’, and ‘Orochi’, at GARES. Although wild accessions of wasabi *E. japonicum* were limited in the current and previous studies, the phylogenetic analysis suggested that Ulleung Island wasabi accessions may represent different escaped cultivars.

We found that deviation in the frequency of codon usage among the 14 wild and cultivated wasabi was mainly observed in the Ulleung Island Dodong accession. In the case of stop codon usage of the three accessions (Dodong, Kicheongsan, and ‘Daewang’), there was deviation from the remaining wild and cultivated wasabis. It seemed that stop codon usage deviation was limited to the lineage that included two Ulleung Island wasabi (Dodong and Kicheongsan) and one major cultivar, ‘Daewang’, based on the plastome tree topology (Figure 6). Nonetheless, other accessions belonging to the same lineage, such as ‘Shogun’, Chusan, ‘Green Thumb’, ‘Micado’, and ‘Orochi’, displayed stop codon usage similar to the wild *Eutrema* species and three major wasabi cultivars from Japan.

## 3. Discussion

### 3.1. Taxonomic Identity of Ulleung Island Wasabi and Its Relationships to Wild and Cultivated Wasabis Inferred by Complete Chloroplast Genomes

In the present study, we determined, for the first time, the taxonomic identity of Ulleung Island wasabi based on complete plastome sequences. Historically, *Wasabia koreana*, coined with the common name Korean wasabi, is known to occur on Ulleung Island, Korea [34]. However, the distribution of wasabi species in Korea and the nomenclature and local name of *W. koreana* have been controversial since the late 1950s [29]. A careful re-examination of the type specimen of *W. koreana* revealed that the taxon coined with *W. koreana* is actually *Cardamine pseudowasabi* and that *E. japonicum* on Ulleung Island has long been misidentified as *W. koreana* [29]. An important question regarding whether *E. japonicum* on Ulleung Island is native was inappropriately addressed based on wasabi cultivars in Korea only [31]. Randomly amplified polymorphic DNA (RAPD) markers [31] showed no clear clustering patterns in Ulleung Island wasabi, highlighting the ineffectiveness of this type of marker for wasabi identification. Our current study shows that Ulleung Island wasabi accessions are most likely to be escaped cultivars, and they represent at least three different cultivar genotypes. Three putative wild accessions of Ulleung Island wasabi turned out to be ‘Shogun’ and ‘Green Thumb’-‘Micado’-‘Orochi’ genotype cultivars. Although limited number of wild accessions of *E. japonicum* from Japan were available for their plastomes, the phylogenetic relationships between the wild populations and cultivated plants suggested that Ulleung Island wasabi accessions are most likely to be escaped cultivars. Another hypothesis is that Ulleung Island wasabi are native and that modern cultivars originated from them. However, this hypothesis seems unlikely because Ulleung Island wasabis are distantly related to wild wasabis (Figure 6), and the occurrence of the three major cultivar types on a very small, geologically young Ulleung Island as progenitors of recent modern cultivars seems highly unlikely. Our results support the view that Ulleung Island wasabis were initially introduced by the Japanese in the 1920s for cultivation but were abandoned and subsequently escaped to natural areas upon the liberation of Korea from Japanese colonial rule in the early 1950s [31]. It is, however, necessary to conduct a much broader population-level sampling on Ulleung Island, assess the degree of genetic variation and population differentiation, and determine their phylogenetic relationships with wild wasabi populations in Japan to reach concrete conclusions on the taxonomic identity of Ulleung Island wasabi.

Due to the long cultivation history of wasabi and the self-selection of agriculturally desired traits by farmers on a local scale, lineage archives for the modern cultivars are sparse [3]. It has been suggested that almost all modern varieties were derived from three major cultivars, ‘Fujidaruma’, ‘Shimane No. 3’, and ‘Mazuma’ [3,15]. ‘Mazuma’, developed in the 1950s, is a widely cultivated wasabi variety originating in Shizuoka Prefecture, Japan. The mother line of ‘Fujidaruma’ is believed to be the old cultivar ‘Daruma’, which is considered to be the descendant of the first domesticated wasabi in Shizuoka Prefecture, about 400 years ago [15]. The ‘Shimane No. 3’ cultivar is a natural hybrid between the semidomesticated native variety ‘Shimane zairai’ from Shimane Prefecture and semi-domesticated native variety ‘Hanbara’ from Tokyo, and based on the complete plastome sequences, the mother line is ‘Hanbara’ [3]. This study provides additional maternal lineage based on phylogenetic relationships among modern-day cultivars (Figure 6). For example, the ‘Mazuma’ cultivar genotype shared its most recent mother line common ancestor with the ‘Daruma’ cultivar genotype maintained in Korea. One major mother line seemed to be behind the origin of several modern cultivars, such as ‘Shogun’, ‘Green Thumb’, ‘Micado’, and ‘Orochi’. In addition, the ‘Shimane No. 3’ cultivar genotype is more closely related to the ‘Fujidaruma’ cultivar than to the ‘Mazuma’. It is necessary to obtain nuclear genomic data to fully unravel the complex asexual and sexual breeding histories of modern wasabi cultivars to further verify these organelle phylogenomic relationships.

Although chloroplast genomes are highly conserved among cultivated wasabi, this study identified limited yet informative chloroplast DNA regions that could be used to identify the mother line of modern-day cultivars (Supplementary Table S2). As shown earlier, two major chlorotypes among major wasabi cultivars are supported by numerous types of variations (point mutations, indels, mononucleotide repeats, and inversions) across the entire chloroplast genomes; one type includes ‘Mazuma’-‘Daruma’ and the other includes the remaining cultivars (two major cultivars in Japan, five major cultivars at GARES, and three samples from Ulleung Island). Three regions, *trnH*-GUG/*psbA* (10T’s/9T’s), *trnK*-UUU/*rps16* (8A’s/C8A’s), and *psbZ*/*trnG*-GCC (C/A), are variable sites that can be used to distinguish ‘Mazuma’ from ‘Daruma’ cultivars. There are only two regions (C/T in *rpoC2* and 10T’s/9’s in *rpl32*/*trnL*-UAG) that have distinguished between ‘Fujidaruma’ and ‘Shimane No. 3’, two of three major modern cultivars in Japan. The ‘Mazuma’ and any cultivars developed from this mother line can be easily distinguished by numerous chloroplast DNA markers from the two other major cultivars, ‘Fujidaruma’ and ‘Shimane No. 3’. The chlorotype of “Dodong and Shogun” based on their identical plastome sequences can be distinguished from the chlorotype of “Micado and Orochi” by three chloroplast regions: T/C in *rpoC2*, 13T’s/14T’s in *rps12*/*trnV*-GAC, and 9T’s/10T’s in *rpl32*/*trnL*-UAG. Chusan wasabi chlorotype contains one point mutation “G” in 23S rRNA. Lastly, the chlorotype of “Kicheongsan-Daewang-Green Thumb” based on their identical plastome sequences can be distinguished from the “Dodong-Shogun” type by one point mutation (T/C) in *rpoC2* and one mononucleotide repeat (9 T’s/10 T’s in *rpl32*/*trnL*-UAG). Although chloroplast markers appear to be effective in discriminating among different cultivars based on maternal lineage history, there is an urgent need to develop highly variable nuclear genome-based markers, for example, genotyping-by-sequencing [35], MIG-Seq [36], and RAD-seq [37] for breeding, barcoding, and conservation [3].

### 3.2. Plastome Divergence Hotspots in Wild and Cultivated Wasabi E. japonicum

In this study, we identified five divergence hotspots in the cp genomes of wild and cultivated wasabi. These regions include four intergenic spacers (*rbcL*/*accD*, Pi = 0.00435; *trnW*-CCA/*trnP*-UGG, Pi = 0.00291; *psbT*/*psbN*, Pi = 0.00218; *psbZ*/*trnG*-GCC, Pi = 0.00201) and one genic region (*ycf1*, Pi = 0.00218), which could be useful as molecular markers for species identification and the infraspecific phylogeographic study of wasabi. These *Eutrema* hotspot regions can be compared to those of other Brassicaceae congeneric species. For the six species of Triangle U *Brassica* species and closely related two *Sinapis* species (Brassicaceae), five mutation hotspots were identified for DNA barcoding markers and phylogenetic analysis: *psbA*, *matK*/*rps16*, *rpl32*, *rpl32*/*trnL*, and *ycf1* [38]. For the six species of *Orychophragmus* in Brassicaceae [39], nine intergenic spacers were identified as divergence hotspots: *trnH*/*psbA*, *atpI*/*rps2*, *trnM*/*atpE*, *atpB*/*rbcL*, *ndhC*/*trnV*, *accD*/*PsaI*, *petB*/*petD*, *rpl23*/*trnL*, and *ndhE*/*ndhG*. Divergence hotspots in plastomes are not highly conserved in congeneric species across members of the family Brassicaceae. However, at least for genic regions, *ycf1* shows high sequence divergence, which has value for the phylogenetic analysis of *Eutrema* and related genera, as well as other angiosperms (e.g., [40,41,42,43,44]). In addition to identifying divergence hotspot regions in the sliding window analysis (Figure 5), highly variable regions among cultivars—*trnH*-GUG/*psbA* (five sites), *rpl32*/*trnL*-UAG (three sites), *clpP* intron 1 (three sites), and *trnK*-UUU/*rps16* (three sites)—can be effective chloroplast DNA markers for population genetic and phylogeographic studies of *Eutrema*.

In conclusion, we sequenced the complete chloroplast DNA sequences of three naturally occurring Ulleung Island wasabi plants and six cultivars from GARES in Korea and determined their taxonomic identity and phylogenetic relationships with wild *Eutrema* species in East Asia and three major cultivars from Japan. It is probable that the three accessions from Ulleung Island are escaped cultivars, and eight chlorotypes have been identified for the major cultivars and the Ulleung Island wasabi. Additionally, we identified different types of variations that distinguish different cultivar lines and five highly divergent hotspot regions in the chloroplast genome.

## 4. Materials and Methods

### 4.1. Plant Sampling, DNA Extraction, and Plastome Sequencing: Assembly and Annotation

We sampled three accessions of Ulleung Island wasabi, two putative wild accessions, Dodong (DD) and Chusan (CS), and one wild origin cultivated accession (Kicheongsan) at the Kicheongsan Botanical Garden, Pohang, Korea (Figure 1). Of the four commonly known localities of putative wild wasabi populations on Ulleung Island, we sampled two wild accessions along the streams representing two major geographical ranges of wasabi (northern and southeastern part of the island). We also sampled six major wasabi cultivars maintained at Gangwondo Agricultural Research and Extension Services (GARES; Taebaek-si, Gangwon-do Province, Republic of Korea): ‘Daruma’, ‘Shogun’, ‘Green Thumb’, ‘Daewang’ (unnamed cultivar from the Daio Wasabi Farm, Japan), ‘Micado’, and ‘Orochi’. Total DNA was extracted from one individual per accession using the DNeasy Plant Mini Kit (Qiagen, Carlsbad, CA, USA). A DNA library was constructed using the TruSeq Nano DNA Kit with a protocol according to the Sample Preparation Guide provided by the manufacturer (Macrogen Inc., Seoul, Republic of Korea). Genome sequencing was performed on the Illumina Hi-Seq 4000 (Illumina, Inc., San Diego, CA, USA) platform with 151 bp read size and paired-end type (Macrogen Inc.). The resulting paired-end reads were assembled de novo using Velvet v1.2.10, with multiple k-mers with coverage ranging from 293 to 4130 [45]. The tRNAs were confirmed using tRNAscan-SE [46], and the sequences were annotated using Geneious R10 [47] and deposited in GenBank. Annotated sequence files in the GenBank were used to draw a circular map with OGDRAW v1.2 [48].

### 4.2. Comparative Plastome Analysis

Using the Shuffle-LAGAN mode [49] of mVISTA [32], 14 complete plastomes of *E. japonicum*, including wild Japanese wasabi (Hokkaido and Ishikawa), major Japanese wasabi cultivars (‘Fujidaruma’, ‘Mazuma’, and ‘Shimane No. 3’), and three Ulleung Island wasabi (Chusan, Dodong, Kicheongsan) and six cultivars currently available in Korea (‘Daewang’, ‘Daruma’, ‘Green Thumb’, ‘Micado’, ‘Orochi’, and ‘Shogun’) were compared. Fourteen *E. japonicum* plastomes were aligned using the back-translation approach with MAFFT ver. 7 [50], and manually edited with Geneious R10 [47]. Using DnaSP 6.10 [33], sliding window analysis with a step size of 200 bp and window length of 800 bp was performed to determine the nucleotide diversity (Pi, π) of the plastomes. The codon usage frequency was calculated using MEGA 7 [51] based on the relative synonymous codon usage (RSCU) value [52], which is a simple measure of nonuniform usage of synonymous codons in a coding sequence. DNA code used by bacteria, archaea, prokaryotic viruses, and chloroplast proteins were also used [53].

### 4.3. Phylogenetic Analysis

We selected twenty-four accessions, including four outgroup species (*E. yungshunense*, NC058526; *E. botschantzevii*, NC29379; *E. salsugineum*, NC028170; and MK637715), based on previous phylogenetic analyses and available *Eutrema* plastome sequences in GenBank [3]. The ingroup included previously reported accessions, i.e., *E. deltoidum* (NC073149), *E. heterophyllum* (NC028728), *E. yunnanense* (NC028727), yuriwasabi *E. tenue* (LC500907 and LC500908), wild and wild wasabi variety *E. japonicum* (LC500902 and LC500903), and wasabi cultivars (‘Mazuma’, LC500901; ‘Fujidaruma’, LC500900; ‘Shimane No. 3’, LC500906). Lastly, the ingroup accessions included three newly sequenced putatively wild *E. japonicum* from Ulleung Island, Korea and six cultivars (‘Daewang’, ‘Daruma’, ‘Green Thumb, ‘Micado’, ‘Orochi’ and ‘Shogun’) from Gangwondo Agricultural Research and Extension Services (GARES). The sequences were aligned using MAFFT v7 [50] in Geneious R10 [47] and maximum likelihood (ML) analysis based on the best-fit model of “TVM+F+R3” was conducted using IQ-TREE 1.4.2 [54]. A nonparametric bootstrap analysis with 1000 replicates was performed.

## Figures and Tables

**Table 1:** Summary of the characteristics of the three Ulleung Island wasabi and six major cultivars in Korea.

Taxa	Ulleung Island			Major Cultivars in Korea (GARES *)					
	Kicheongsan	Chusan	Dodong	‘Daewang’	‘Daruma’	‘Green Thumb’	‘Micado’	‘Orochi’	‘Shogun’
Total cpDNA size (bp)	153,851	153,851	153,852	153,851	153,794	153,851	153,849	153,849	153,852
GC content (%)	36.4%	36.4%	36.4%	36.4%	36.4%	36.4%	36.4%	36.4%	36.4%
LSC size (bp) /GC content (%)	83,889/34.1%	84,006/34.1%	84,006/34.1%	84,006/34.1%	83,990/34.0%	84,006/34.1%	84,006/34.1%	84,006/34.1%	84,006/34.1%
IR size (bp) /GC content (%)	26,017/42.5%	26,017/42.5%	26,017/42.5%	26,017/42.5%	26,007/42.5%	26,017/42.5%	26,016/42.5%	26,016/42.5%	26,017/42.5%
SSC size (bp) /GC content (%)	17,811/29.4%	17,811/29.4%	17,812/29.4%	17,811/29.4%	17,790/29.4%	17,811/29.4%	17,811/29.4%	17,811/29.4%	17,812/29.4%
Number of genes	131	131	131	131	131	131	131	131	131
Number of protein-coding genes	84	84	84	84	84	84	84	84	84
Number of tRNA genes	37	37	37	37	37	37	37	37	37
Number of rRNA genes	8	8	8	8	8	8	8	8	8
Number of duplicated genes	17	17	17	17	17	17	17	17	17
Accession Number	PP413704	PP413705	PP413706	PP413707	PP413708	PP413709	PP413710	PP413711	PP413712

* GARES: Gangwondo Agricultural Research and Extension Services. LSC: Large single copy region, IR: Inverted repeat, SSC: Small single copy region.

## Data Availability

The data presented in this study are publicly available in NCBI GenBank (https://www.ncbi.nlm.nih.gov/) (accession numbers: PP413704-PP413712).

## Supplementary Materials

The following supporting information can be downloaded from https://www.mdpi.com/article/10.3390/genes15040457/s1, Table S1: Codon usage and codon-anticodon recognition pattern for tRNA in the 14 *E. japonicum* plastomes. Table S2: Variable sites in chloroplast genomes of Ulleung Island wasabi accessions, three major Japanese cultivars, and six cultivars maintained at GARES, Korea. Inversions are indicated in red.
